# A Case of Pneumonia with Cerebral Complications

**Published:** 1898-02-12

**Authors:** Sidney Coupland

**Affiliations:** Physician to the Hospital


### MEDICAL PROGRESS AND HOSPITAL CLINICS.

[*The Editor will be glad to receive offers of co-operation and contributions from members of the profession. All letters should be addressed to* THE EDITOR, AT THE OFFICE, 28 & 29, SOUTHAMPTON STREET, STRAND, LONDON, W.C.]

### A CASE OF PNEUMONIA WITH CEREBRAL COMPLICATIONS.

A Clinical Lecture given at the Middlesex Hospital by SIDNEY COUPLAND, M.D., F.R.C.P., Physician to the Hospital.

Gentlemen,—I propose to-day to relate to you the particulars of a case of pneumonia, the subject of which is now in Hertford Ward, convalescing from an attack that was characterised by an unusual feature. The patient is a man 34 years of age, fairly robust and well-developed, whose attack commenced in the characteristic abrupt fashion of acute pneumonia, on the third day of which he was admitted into the hospital. I will read you the notes of the case, and then make such comments upon it as may be required, for although at first sight the case may not seem to be worthy of detailed study, you will, I hope, before I close, feel, with me, that it has some important bearings upon the correct interpretation of the phenomena of this extremely interesting and remarkable disease.

[*We omit these notes, as the whole story is well told in Dr. Coupland's interesting comments.*]

When this patient was admitted his case presented no unusual features. It exhibited the well-marked characters of acute lobar pneumonia in a strong and healthy man, whose habits, so far as known to us, had not been markedly intemperate. It is true that we subsequently learnt that he had been a free drinker, but he was never a drunkard; nor did he manifest any of the features of a pronounced alcoholic. Nor could the case be regarded as an exceptionally severe one. He came in on the third day of the attack, with a temperature of 103 8, and a pulse-rate of 120, the pulse-respiration ratio being very nearly as 1:2. I may remark incidentally that such a high rate of respiration is almost characteristic of acute pneumonia. It may be reached in acute tuberculosis, or even exceeded in neurotic states; but, these apart, its occurrence is nearly distinctive.

The seat of pain determined the locality of the inflamed lung. The pain, which was the first symptom complained of, was more persistent than usual, and doubtless denoted a considerable amount of concomitant pleurisy. But that pneumonia rather than pleurisy was the predominant affection was further proved by the results of physical examination and by the scanty, viscid, rusty coloured sputa he expectorated. The examination gave evidence of consolidation of the lower two-thirds of the lower lobe of the right lung, the fine pneumonic crepitation proving that the stage of exudation had not ceased. For three days his illness pursued an uneventful course. The physical signs showed that the pneumonic consolidation was extending, but it never involved the upper lobe of the lung, and there were no signs of implication of the left lung. By October 16th (the sixth day) its area was well confined to the right lower and middle lobes. The signs were never quite typical, for the presence of tubular breathing (perhaps the most definite mark of lung-consolidation) was not widely distributed. At the most it was not to be heard beyond a small area in the vicinity of the angle of the scapula, and on some days even that was absent, and only weakened and faint breath-sounds could be heard on auscultation over the lung. This fact, coupled with the observation of an œgophonic vocal resonance, might well have led to the suspicion that pleural effusion rather than pneumonic consolidation was the actual condition present. On this I may remark that I have no doubt there was a fair amount of pleurisy, that the condition was, strictly speaking, pleuro-pneumonia, but that there could be no such large effusion as would account for the area of dulness is certain, because of the following facts: (1) The line of the upper limit of dulness was oblique, and followed very closely the limit of the lower lobe of the lung; (2) the presence of crepitation at the base of the lung; (3) the persistence of (and, later, actual intensification of) the tactile vocal fremitus; (4) the maintenance of the apex-beat of the heart in the normal situation; and (5) the well-marked rusty sputa, which are characteristic of pneumonia.

You will often find that bronchial or tubular breathing is absent over part or the whole of a consolidated lung, although the physical condition would lead to its production. This is explained by the fact that the fibrinous exudation in pneumonia may block the bronchioles so effectually as actually to prevent the passage of the sound-vibrations being conducted to the surface. We did, however, occasionally hear this type of breath-sound, and that goes to support the conclusion, since from time to time some of these channels might have been freed of their obstruction.

It is to be noted further that the fever did not run a severe course. At no time did the temperature exceed 103·8 deg., and there was generally a morning remission of 2 deg. to 3 deg.—an unusual thing in pneumonia, and not to be accounted for in this case by the mild antipyretic measure of cold sponging of the body resorted to when the temperature rose above 103 deg. There had been no complaint of headache, and up to the sixth day his nights had been fairly quiet and sleep undisturbed.

The night of the sixth-seventh day (*i.e.*, Saturday-Sunday, October 16th-17th) he became delirious, the delirium being active, but not noisy; he got out of bed, and passed a most restless night. The next morning, the seventh day, when mostly the crisis of pneumonia supervenes, he seemed flushed and excited, and there was slight tremor of the tongue and hands; but in no other respect did his symptoms resemble those of delirium tremens. Indeed, he was not then delirious, but answered questions rationally. I was surprised to find that the expectoration, although very scanty, presented still the bright, rusty character, and I feared that the inflammation was still spreading. For rusty expectoration corresponds with the earlier congestive stage of pneumonia, and perhaps to a slight extent to that also of red hepatisation. Yet physical examination did not reveal any further extension of dulness, nor signs of involvement of the left lung, although the presence of rhonchi on that side showed that there was some bronchial congestion—" collateral hyperæmia," as it used to be called.

(*To be continued.*)

